# Genome Sequences of a Novel Strain of Big Cypress Orbivirus Isolated from a Dead Florida White-Tailed Deer (Odocoileus virginianus)

**DOI:** 10.1128/MRA.01717-18

**Published:** 2019-03-14

**Authors:** Mohammad Shamim Ahasan, Juan M. Campos Krauer, Kuttichantran Subramaniam, John A. Lednicky, Julia C. Loeb, Katherine A. Sayler, Samantha M. Wisely, Thomas B. Waltzek

**Affiliations:** a Department of Infectious Diseases and Immunology, College of Veterinary Medicine, University of Florida, Gainesville, Florida, USA; b Emerging Pathogens Institute, University of Florida, Gainesville, Florida, USA; c Faculty of Veterinary and Animal Sciences, Hajee Mohammad Danesh Science and Technology University, Dinajpur, Rangpur, Bangladesh; d Department of Large Animal Clinical Sciences, College of Veterinary Medicine, University of Florida, Gainesville, Florida, USA; e Department of Wildlife Ecology and Conservation, University of Florida, Gainesville, Florida, USA; f Department of Environmental and Global Health, College of Public Health and Health Professions, University of Florida, Gainesville, Florida, USA; Georgia Institute of Technology

## Abstract

Here, we report the coding sequences of Big Cypress orbivirus isolated from a dead white-tailed deer (Odocoileus virginianus) from Florida in 2017. To our knowledge, this is the first detection of Big Cypress orbivirus in a vertebrate host.

## ANNOUNCEMENT

The genus *Orbivirus* within the family *Reoviridae* includes arboviruses with nonenveloped nucleocapsids composed of outer and inner proteinaceous layers that surround a segmented, double-stranded RNA (dsRNA) genome (1). The genes of the structural proteins of the outer capsid layer (VP2 and VP5) typically exhibit the greatest genetic variability and determine the orbivirus serotype. By comparison, the genes of the inner capsid structural proteins (VP3 coding the T2 protein) display less genetic variability and are used for species demarcation (1).

In September of 2017, a farmed white-tailed deer from Liberty County, Florida, was observed lethargic and then found dead 5 days later. A necropsy was performed on the fresh carcass, and selected specimens were submitted to the University of Florida Cervidae Health Research Initiative for evaluation. A 10% (wt/vol) spleen tissue homogenate was generated in advanced Dulbecco’s modified Eagle’s medium (Invitrogen Corp.) supplemented with 2 mM l-alanyl-l-glutamine (GlutaMAX, Invitrogen Corp.) and antibiotics (50 µg/ml penicillin, 50 µg/ml streptomycin, and 100 µg/ml neomycin [PSN; Invitrogen Corp.]) using a manual tissue grinder (Fisher Scientific, Waltham, MA, USA). The tissue homogenate was then filtered through a sterile 0.45-µm filter to remove particulates and contaminating bacteria and fungi and stored at –80°C. The homogenate was inoculated onto Aedes albopictus clone C6/36 cells where virus-induced cytopathic effects were observed 5 days postinoculation.

Viral RNA was extracted from virions in spent cell culture medium using a QIAamp viral RNA minikit (Qiagen) and served as the template for the construction of a cDNA sequencing library using a NEBNext Ultra RNA library prep kit. The resulting cDNA library was sequenced on a MiSeq (Illumina, San Diego, CA) instrument using a v3 600-cycle kit. After filtering the low-quality reads and quality trimming in CLC Genomics Workbench using default parameters, a total of 1,824,518 reads were obtained with an average read length of 237 bp. Following the removal of host sequences (Aedes albopictus; GenBank accession number MNAF00000000) using Kraken (2), a *de novo* assembly of 147,263 paired-end reads was performed in SPAdes (3). A total of 31 assembled contigs (*N*_50_ value, 743 bp) were screened by BLASTX in CLC Genomics Workbench using a custom virus database created from virus protein sequences retrieved from the UniProt Knowledgebase (https://www.uniprot.org/uniprot/). The BLASTX analysis identified all 10 segments of an orbivirus closely related to Big Cypress orbivirus (BCPOV) previously isolated in C6/36 cells from Psorophora columbiae mosquitoes collected in Big Cypress National Preserve (BCNP-2-151) in southern Florida (4). The total length of the nearly complete coding sequences of the 10 BCPOV strain OV624 (BCPOV-OV624) segments was 17,751 bp, with a G+C content of 41.73%. The integrity of the BCPOV-OV624 coding sequences was verified by mapping the reads using Bowtie 2 (5) and inspecting the alignments in Tablet 1.17.08.17 (6). The average coverage of the BCPOV-OV624 genome was 1,699 reads/nucleotide. The BCPOV-BCNP-2-151 displayed a similar nearly complete coding sequence length of 17,854 bp and a G+C content of 41.53% (4). The untranslated regions of the 10 segments of BCPOV-OV624 were not determined, and only the partial coding sequence for the VP2 protein was determined.

**FIG 1:**
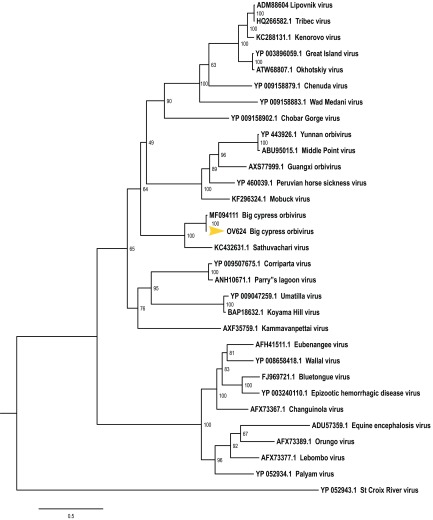
Maximum likelihood phylogram, based on the amino acid sequence alignment of the T2 proteins, depicting the relationship of Big Cypress orbivirus strain OV624 to 30 orbiviruses. Bootstrap values are given at each node. Branch lengths represent the number of inferred substitutions, as indicated by the scale.

The inner (T2; used to determine orbivirus species) and outer capsid (VP2; used to determine orbivirus serotype) proteins of BCPOV-OV624 showed 99% and 35% amino acid identity, respectively, compared with BCPOV-BCNP-2-151. Thus, BCPOV-OV624 represents a new BCPOV strain and likely a new serotype. Maximum likelihood phylogenetic analysis performed in IQ-TREE (7), based on the amino acid sequence alignment of the T2 proteins from 30 orbiviruses, supported BCPOV-OV624 as the closest relative to BCPOV-BCNP-2-151 (Fig. 1).

BCPOV has been proposed as the prototype of a new orbivirus species (4). The isolation of BCPOV-OV624 from a dead white-tailed deer suggests it may represent a previously unknown mosquito-borne cervid pathogen. Further study is needed to determine the vertebrate host range of BCPOV, including its potential role in disease among farmed and wild white-tailed deer populations.

### Data availability.

The genome sequences for BCPOV-OV624 and raw sequence data have been deposited in GenBank (accession numbers MK105769 to MK105778) and the Sequence Read Archive (accession number PRJNA508942).

## References

[1] MacLachlan NJ 2017 Chapter 15—Reoviridae p 299–317. *In* Dubovi EJ (ed), Fenner's veterinary virology, 5th ed. Academic Press, Boston, MA.

[2] Wood DE, Salzberg SL 2014 Kraken: ultrafast metagenomic sequence classification using exact alignments. Genome Biol 15:R46. doi:10.1186/gb-2014-15-3-r46. 24580807 PMC4053813

[3] Bankevich A, Nurk S, Antipov D, Gurevich AA, Dvorkin M, Kulikov AS, Lesin VM, Nikolenko SI, Pham S, Prjibelski AD, Pyshkin AV, Sirotkin AV, Vyahhi N, Tesler G, Alekseyev MA, Pevzner PA 2012 SPAdes: a new genome assembly algorithm and its applications to single-cell sequencing. J Comput Biol 19:455–477. doi:10.1089/cmb.2012.0021. 22506599 PMC3342519

[4] Sadeghi M, Popov V, Guzman H, Phan TG, Vasilakis N, Tesh R, Delwart E 2017 Genomes of viral isolates derived from different mosquitos species. Virus Res 242:49–57. doi:10.1016/j.virusres.2017.08.012. 28855097 PMC5665172

[5] Langmead B, Salzberg SL 2012 Fast gapped-read alignment with Bowtie 2. Nat Methods 9:357. doi:10.1038/nmeth.1923. 22388286 PMC3322381

[6] Milne I, Bayer M, Cardle L, Shaw P, Stephen G, Wright F, Marshall D 2010 Tablet—next generation sequence assembly visualization. Bioinformatics 26:401–402. doi:10.1093/bioinformatics/btp666. 19965881 PMC2815658

[7] Nguyen LT, Schmidt HA, von Haeseler A, Minh BQ 2015 IQ-TREE: a fast and effective stochastic algorithm for estimating maximum-likelihood phylogenies. Mol Biol Evol 32:268–274. doi:10.1093/molbev/msu300. 25371430 PMC4271533

